# Letter of A. M. Barker, M. D., Secretary

**Published:** 1881-09

**Authors:** 


BUFFALO, N. Y., August 25, 1881.

*To the Editors of the Buffalo Medical and Surgical Journal:*

At the semi-annual meeting of the Erie County Medical Society, the Board of Censors presented a report regarding the operation of the new medical law, in which they recommended "that the secretary of the Society be directed to notify all members to send the names of persons known to them to practice medicine in this county in violation of the new medical law, to the Board, and also report the names of druggists who are known to prescribe illegally and deal out medicines to patients in their stores."

Will you kindly, through the columns of your journal, call the attention of the members to the recommendation of the committee, and request them to send any information they may have concerning the matter to Dr. Edward Storck, Buffalo, N. Y.

A. M. BARKER, M. D., Secretary.

